# An oral preparation of *Lactobacillus acidophilus* for the treatment of uncomplicated acute watery diarrhoea in Vietnamese children: study protocol for a multicentre, randomised, placebo-controlled trial

**DOI:** 10.1186/1745-6215-14-27

**Published:** 2013-01-28

**Authors:** Marion-Eliëtte Kolader, Ha Vinh, Pham Thi Ngoc Tuyet, Corinne Thompson, Marcel Wolbers, Laura Merson, James I Campbell, Tran Thi Ngoc Dung, Ha Manh Tuan, Nguyen Van Vinh Chau, Jeremy Farrar, H Rogier van Doorn, Stephen Baker

**Affiliations:** 1 The Hospital for Tropical Diseases, Wellcome Trust Major Overseas Programme, Oxford University Clinical Research Unit, Ho Chi Minh City, Vietnam; 2 Children’s Hospital 2, Ho Chi Minh City, Vietnam; 3 The Hospital for Tropical Diseases, Ho Chi Minh City, Vietnam; 4 Department of Medical Microbiology, Academic Medical Centre, Amsterdam, the Netherlands; 5 Nuffield Department of Clinical Medicine, Oxford University, Oxford, UK; 6 The London School of Hygiene and Tropical Medicine, London, UK

**Keywords:** Probiotics, *Lactobacillus* spp., Diarrhoea, Randomised controlled trial, Rotavirus, Norovirus

## Abstract

**Background:**

Diarrhoeal disease is a major global health problem, particularly affecting children under the age of 5 years. Besides oral rehydration solution, probiotics are also commonly prescribed to children with acute watery diarrhoea in some settings. Results from randomised clinical trials (RCTs) in which investigators studied the effect of probiotics on diarrhoeal symptoms have largely shown a positive effect; yet, the overall quality of the data is limited. In Vietnam, probiotics are the most frequently prescribed treatment for children hospitalised with acute watery diarrhoea, but there is little justification for this treatment in this location. We have designed a RCT to test the hypothesis that an oral preparation of *Lactobacillus acidophilus* is superior to placebo in the treatment of acute watery diarrhoea in Vietnamese children.

**Methods:**

This RCT was designed to study the effect of treatment with *L. acidophilus* (4 × 10^9^ colony-forming units/day) for 5 days for acute watery diarrhoea against a placebo in 300 children ages 9 to 60 months admitted to hospitals in Vietnam. Clinical and laboratory data plus samples will be collected on admission, daily during hospitalisation, at discharge, and at follow-up visits for a subset of participants. The primary end point will be defined as the time from the first dose of study medication to the start of the first 24-hour period without diarrhoea as assessed by the on-duty nurse. Secondary endpoints include the time to cessation of diarrhoea as recorded by parents or guardians in an hourly checklist, stool frequency over the first 3 days, treatment failure, rotavirus and norovirus viral loads, and adverse events.

**Discussion:**

The existing evidence for the use of probiotics in treating acute watery diarrhoea seems to favour their use. However, the size of the effect varies across publications. An array of different probiotic organisms, doses, treatment durations, study populations, designs, settings, and aetiologies have been described. In this trial, we will investigate whether probiotics are beneficial as an adjuvant treatment for children with acute watery diarrhoea in Vietnam, with the aim of guiding clinical practice through improved regional evidence.

**Trial registration:**

Current Controlled Trials ISRCTN88101063

## Background

Diarrhoeal disease is a global health issue, yet the vast burden is in young children in developing countries [1]. In 2010, more than 7 million children younger than 5 years old died, and 15% of these deaths were attributed to diarrhoea [2,3]. Typically, diarrhoeal episodes are self-limiting, and patients often recover without receiving an aetiological diagnosis. In those who are diagnosed, rotavirus is the most frequently identified pathogen, followed by a multitude of other viral, parasitic and bacterial agents [4]. Vietnam is a rapidly developing country in Southeast Asia, with an estimated mortality rate of 23 per 1,000 live births among children younger than 5 years old [2]. The number of deaths in children in this key age bracket in Vietnam in 2010 was 34,940, 11% of which were attributable to diarrhoeal infections [3].

Oral rehydration solution (ORS), zinc and antimicrobials are the main treatments for acute diarrhoea in children in Vietnam [5]. During a cross-sectional study of approximately 1,500 children hospitalised with diarrhoea, however, we found that 11% of diarrhoeal patients were given probiotics prior to hospitalisation, and 71% of children were prescribed probiotics during hospitalisation (Phan Vu Tra My *et al.*, unpublished manuscript). Probiotics are preparations of bacteria and yeasts that are considered to confer a beneficial health effect when taken in an adequate amount [6]. Probiotics have been studied extensively for their effects in preventing and treating a multitude of conditions, including the treatment of lactose intolerance, traveller’s diarrhoea and the prevention and treatment of nosocomial diarrhoea [7-10]. In acute diarrhoea, a reduction in the frequency of diarrhoeal symptoms has been reported in adults and children treated with probiotics. This cessation of diarrhoeal symptoms has been observed specifically in patients with rotavirus, in those treated during the early stages of a disease episode, and in those treated with *Lactobacillus* spp. [11-14]*.* Investigators in previous trials have evaluated the effect of probiotic colonisation on rotavirus and found a potential reduction in rotavirus-shedding in cohorts of Taiwanese, Polish and Italian children [15-17]. Probiotics are well-tolerated; adverse events (AEs) are rare and generally occur in patients with underlying chronic diseases or in those on immunosuppressive therapy [18,19].

In a Cochrane review of the effect of probiotics for the treatment of acute watery diarrhoea, Allen *et al.* combined data from 8,014 participants in 63 studies [20]. The authors noted extensive heterogeneity in study design, definitions, infecting agents, probiotic organisms and dosage. Notwithstanding these caveats, the meta-analysis found probiotics to be effective in reducing the duration of diarrhoea by a mean of 24.76 hours (95% confidence interval (CI) = 15.9 to 33.6 hours) compared to patients who either did not receive probiotics or were given a placebo. On the basis of these same analyses, probiotic therapy was found to reduce the frequency of stools on the second day of treatment by a mean of 0.8 stools (95% CI = 0.45 to 1.14), and it reduced the risk of developing persistent diarrhoea by 59% (95% CI for risk ratio = 0.32 to 0.53). `The authors recommended the design of larger, more robust trials specifically focusing on pathogen identification and the incorporation of standard definitions and end points to inform clinical guidelines.

There is currently no international regulatory agreement for the manufacturing or the clinical use of probiotics, and there are different levels of scientific evidence required to substantiate the health benefits of probiotics [21-23]. This evidence is dependent on the product, the specific disease effects, the strain and the dose. Additionally, probiotics can be classified as either food additives or drugs, depending on the local regulatory authority. This discrepancy has led to interchangeable and often nonstandardised therapeutic use. Therefore, trials incorporating the recommendations from Allen *et al.*[20] to investigate the effects of probiotics for acute diarrhoea are vital to increase the evidence base for the use of probiotics as a clinical therapy in similar settings.

## Methods/design

### Study aims

We hypothesise that *L. acidophilus* is superior to no *L. acidophilus* in the treatment of children admitted to the hospital for acute watery diarrhoea in Ho Chi Minh City, Vietnam. We will test this hypothesis by comparing an oral daily dose of 4 × 10^9^ colony-forming units (CFUs) of *L. acidophilus* against placebo in children hospitalised with acute diarrhoea. The study design presented herein aims to investigate whether probiotic therapy with *L. acidophilus* should be used as an appropriate and efficacious adjuvant to ORS and zinc in treating children hospitalised with acute watery diarrhoea. Using these data, we additionally aim to inform local clinical practice on the use of probiotics for treating acute diarrhoea by improving the quality of clinical evidence.

### Study design

This study is a multicentre, randomised, double-blind, placebo-controlled trial designed to assess the effect of adjunctive probiotic (*L. acidophilus*) therapy in children admitted to the hospital with acute watery diarrhoea. Laboratory endpoints from admission until discharge will be recorded, and colonisation will be assessed in an outpatient follow-up visit with the first 50 subjects who agree to prolonged participation.

### Eligibility

To be eligible for enrolment, patients will need to be between 9 and 60 months of age and require hospitalisation for acute, nonbloody, nonmucoid, watery diarrhoea with a history of less than 3 days, and written informed consent from a parent or guardian will be required. This age group was selected, as healthy children younger than 9 months of age have less solid stools than those in an older age category, making the cessation of diarrhoeal symptoms more difficult to assess. *Diarrhoea* in this study is defined according to the criteria of the World Health Organisation (WHO): the passage of unusually loose or watery stools at least three times within a 24-hour period [5]. According to the Bristol Stool Form Scale, the aspect of the stool will be defined as watery with no solid matter (type 7 stool form) [24].

Patients who meet any of the following criteria will not be eligible for participation in the study: at least one episode of diarrhoeal disease in the month prior to admission, those taking an antiemetic or antidiarrhoeal medication at the time of presentation, those who are known to have short bowel syndrome, those with an underlying chronic inflammatory gastrointestinal disease, those who are immunocompromised or immunosuppressed, those who are on prolonged steroid therapy and those who are severely dehydrated (according to the definitions of the WHO guidelines for the treatment of diarrhoea) [5].

### Inclusion of patients and informed consent

**Figure 1:**
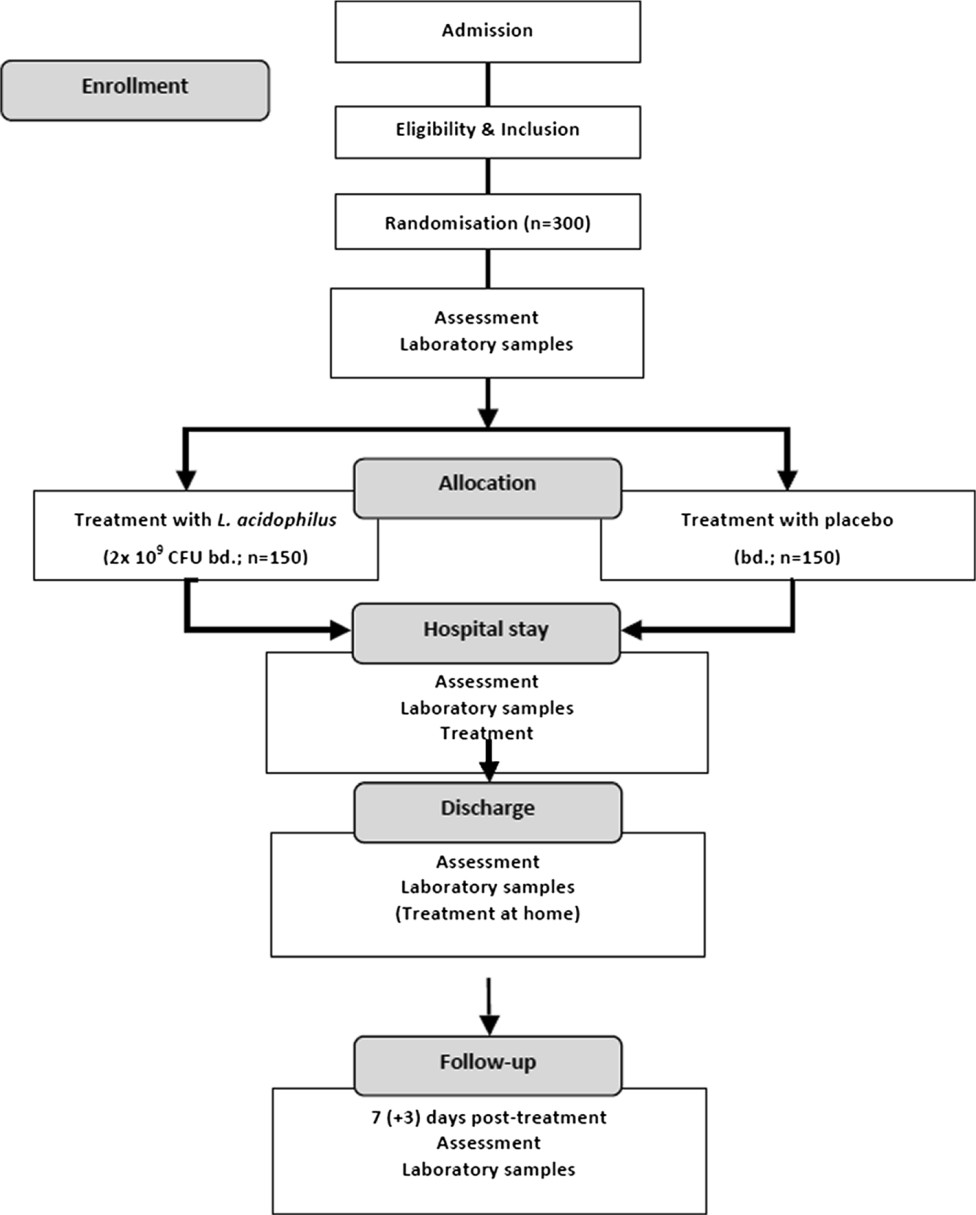
**Trial study flow**-**chart.**

A physician at each participating centre will approach all parents or guardians of patients who meet all the inclusion criteria and none of the exclusion criteria after routine admission. Informed consent will be sought from parents or guardians (Figure 1). If parents or guardians are indecisive about enrolment, they will be given until 72 hours after disease onset to consider entry into the study, after which point the patient will no longer be eligible.

### Study medication and quality control

The study medication (lyophilised *L. acidophilus*, manufactured under the WHO good manufacturing process guidelines) will be purchased from Imexpharm Pharmaceutical Joint Stock Corporation (Cao Lanh, Vietnam). This probiotic is registered with the Vietnam Ministry of Health (MOH) as a drug. The laboratories at Oxford University Clinical Research Unit will perform internal quality control of the medication before the start of the study. Sachets will contain 1 × 10^9^ CFU of *L. acidophilus*. Placebo sachets will contain an excipient (maltodextrin) only. The manufacturer will blind the contents of the sachets, and the master code will be disclosed to the study pharmacist only.

### Treatment groups and duration of treatment

All patients will receive the standard of care, according to the national guidelines for the management of diarrhoeal diseases in children (Vietnam MOH, 2009), supplemented with oral zinc (20 mg per day) for 10 days, according to the WHO guidelines [5]. Subsequently, all patients will be randomly assigned to one of the two study groups at a 1:1 ratio. As probiotics are widely used without adverse effects and scientific publications indicate a variable beneficiary effect, the standard dose of study medication has been increased to avoid underdosing. Therefore, one group of participants will receive twice the standard daily dose of *L. acidophilus* (4 × 10^9^ CFU), and the other group of participants will receive placebo. The regimes will be identical in both study groups: doses every 12 hours for 5 days. For each dose, two sachets of the study medication will be dissolved in 10 ml of water and will be administered orally to participants by their parents or guardians. If the participant vomits within 60 minutes of taking the drug, an additional dose will be given (up to two extra doses in 4 hours). Dosing and redosing will be recorded in the case report form (CRF).

If participants require an antidiarrhoeal or antiemetic medication, they will be categorised as a treatment failure and analysed accordingly. The prescription of any additional treatment, including antimicrobials, will be recorded in the CRF.

If treatment is discontinued because of an AE, the treating physician will record this and monitor the individual until the AE has resolved or stabilised. Participants who discontinue study treatment because of an AE will continue to be followed as per protocol. Physicians will discharge participants after the completion of the 5-day treatment course or 24 hours after the complete cessation of symptoms, whichever occurs first. If participants are discharged before completing the 5-day treatment course, the parents or guardians will be asked to complete the administration of all 10 doses. A follow-up visit at day 12 will be planned with the parents or guardians of participants upon discharge.

### Randomisation and blinding

The study pharmacist will create a computer-generated block randomisation with blocks of varying size and stratification by participating centre. The probiotic and placebo sachets will be prepared in 5-day treatment packages plus vomit doses as described below. These will be labelled in advance and kept in a secure location. Participant numbers, which correspond to a package of sachets, will be assigned to each enrolled patient in strict numerical sequence. The treatment packages will be dispensed according to the participant number once a patient is enrolled into the trial. A chronological log of all enrolled patients will be maintained, and the next available sequential study code will be assigned to patients as they enrol.

### Data collection

Data will be recorded on study-specific CRFs and samples will be collected from each participant as indicated in Table 1. Briefly, after enrolment, study staff will perform the assessments, complete the appropriate CRF sections and collect laboratory samples according to protocol. Study medication will then be assigned. During hospitalisation, daily assessments of the participants will be performed and study staff will collect daily faecal samples or rectal swabs. Once the treating physician has deemed hospitalisation is no longer required, discharge will be arranged, as well as a follow-up visit. On discharge and at follow-up, assessments will be performed and faecal samples collected.

**Table 1:** Clinical and laboratory data collection

	**Admission**	**Daily**	**Discharge**	**Follow-up**
Physical examination	✓	✓	✓	✓
Assessment of dehydration	✓	✓	✓	✓
Assessment of diarrhoea	✓	✓	✓	✓
Data collection	✓	✓	✓	✓
Faecal sample	✓		✓*	✓*
Faecal or rectal swab		✓	✓*	✓*
Blood sample (3 ml) (biochemistry/haematology and viral polymerase chain reaction)	✓			

* If the participant cannot produce a stool sample on discharge, a faecal or rectal swab will be collected instead.

### Sample handling and storage

Samples will be labelled with the patient study code assigned at enrolment. The samples will be used for the purpose of this study, as stated in the protocol, then stored for future use. Consent will be obtained from participants’ parents or guardians for sample storage and/or shipment of specific samples to collaborating institutions for investigations that cannot be performed locally.

### Laboratory methods

During the study, all collected samples will be labelled as stated in the protocol, stored at 4°C and transported to the laboratory at the Oxford University Clinical Research Unit within 24 hours. Routine haematology and biochemistry tests will be performed on blood samples at admission to evaluate the severity of dehydration and disease.

### Bacteriology

Faecal samples will be cultured on blood agar, MacConkey agar, xylose lysine desoxycholate agar, *Campylobacter* selective media and selenite cystine broth (Oxoid Microbiology Products; Thermo Scientific, Basingstoke, UK) to detect *Salmonella* spp.), *Shigella* spp. and *Campylobacter* spp. If pathogenic bacteria are suspected, isolation will be performed on nutrient agar (Oxoid Microbiology Products), followed by identification by standard biochemical reactions and API 20 E (bioMérieux, Marcy l’Etoile, France). Antimicrobial susceptibility testing will be performed on all pathogenic bacteria on Müller-Hinton agar by disk diffusion for ampicillin (10 μg), amoxicillin/clavulanic acid (20/10 μg), ceftriaxone (30 μg), ceftazidim (30 μg), ciprofloxacin (5 μg), nalidixic acid (30 μg), ofloxacin (5 μg), gentamicin (10 μg), chloramphenicol (30 μg) and trimethoprim/sulfamethoxazole (23.75/1.25 μg; Oxoid Microbiology Products). Erythromycin (15 μg) and clindamycin (2 μg) will be added for *Campylobacter* spp. Zone diameters will be interpreted according to the Clinical Laboratory Standards Institute (CLSI) clinical breakpoints. Culture results and antimicrobial susceptibility results will be reported back to the treating clinicians.

### Virology

Norovirus and rotavirus real-time reverse transcriptase polymerase chain reaction (RT-PCR) will be performed on total nucleic acid extracted from blood and faecal samples collected on admission using a previously published method [25]. Results will be reported to the treating clinician and disclosed to the parents or guardians of the participants. Aliquots of faecal samples collected at discharge and during follow-up visits will be stored as 10% vol/vol suspensions in phosphate-buffered saline at −80°C. Faecal or rectal swabs collected during hospitalisation will be stored at −80°C in viral transport media. Norovirus and rotavirus real-time RT-PCR will be performed in batches retrospectively. Because this trial is exploratory, results will be analysed for the patients who test positive for norovirus and/or rotavirus at admission only.

### Primary end point

The primary end point is the time from the first dose of study medication to the start of the first 24-hour period without diarrhoea as assessed by the nurse on duty.

### Secondary end points

The following are the secondary end points:

1. The time from the first dose of study medication to the start of first 24-hour diarrhoea-free period as recorded by parents or guardians in an hourly checklist, which will be collected by the nurses on duty every 12 hours.

2. Treatment failure, defined as the occurrence of (a) No resolution of diarrhoea after 5 days of treatment, (b) severe symptoms for which treatment is stopped, such as the development of severe dehydration, renal failure, (septic) shock, respiratory distress or other syndromes associated with diarrhoeal disease as complications (e.g., pneumonia) [5]; and (c) additional antiemetic (domperidone) or antidiarrhoeal (aluminium silicate or racecadotril) treatment.

3. The frequency of stools during the first 3 days after the first dose of study medication.

4. The load in copies per millilitre of viral transport medium of norovirus and rotavirus measured by RT-PCR, assessed daily from admission until discharge and at follow-up.

5. The duration of hospitalisation, defined as the number of days from hospital admission until discharge.

6. The extent of intestinal *L. acidophilus* colonisation, assessed by analyses of faecal samples or rectal swabs collected on admission, on discharge and at outpatient follow-up for the first 50 participants who agree to participate.

7. The number and severity of AEs.

### Reporting of serious adverse events

All AEs and serious adverse events (SAEs) will be recorded on the participant’s CRF. If a participant experiences a SAE, defined according to International Conference on Harmonisation of Technical Requirements for Registration of Pharmaceuticals for Human Use guidelines on clinical safety data management, the treating physician will inform the principal investigator and complete the specific CRF [26]. All SAEs will be reported as soon as possible to the Data Safety Monitoring Board (DSMB), the research ethics committees of reference and the Hospital Ethical Committee where the participant is admitted.

### Sample size and statistical considerations

Data originating from three hospitals in Ho Chi Minh City suggest that the median duration of hospitalisation in our target population is 5 days (interquartile range = 3 to 6 days; mean and standard deviation [SD] of log_10_ duration = 0.61 and 0.27, respectively) and an approximately normal distribution of the log-transformed data. As we have limited preexisting data on the overall length of diarrhoeal illness (prehospitalised and hospitalised), and as children are usually discharged at the time of resolution of diarrhoea, we used the variability of the length of hospitalisation as the basis of our sample size calculation using R software version 2.15.1. This study is designed to test the hypothesis that probiotic therapy is superior to placebo for the treatment of acute watery diarrhoea. It is powered to detect a 20% decrease in the duration of acute watery diarrhoea (corresponding to an absolute effect size of approximately 24 hours) of twice the standard dose of probiotics compared to placebo. For 80% power with a two-sided 5% significance level, a total of 123 participants per study group are required. To account for potential inadequacies in our assumptions and some loss to follow-up, the sample size was increased by 22%. Therefore, a total sample size of 300 participants (150 in each group) will be recruited.

### Analysis and reporting

All randomised patients will be included in the analysis, following the intention-to-treat principle. The comparison of the primary end point will be performed on a per-protocol population, which excludes participants who are mistakenly randomised (in violation of the inclusion and exclusion criteria) and those who withdraw or are lost to follow-up prior to reaching the primary end point.

The primary end point will be compared between the study arms on the basis of a lognormal accelerated failure time regression model, which models the log-transformed outcome as depending linearly on covariates plus a normally distributed error term for unexplained variation [27]. It generalises the standard linear regression model of the log-transformed outcome to right-censored or interval-censored outcome data that are required for analysing our primary end point. If parents or guardians cannot specify the exact time of cessation of diarrhoea, then the assessment by the nurse on duty who assesses the child every 12 hours will be used instead (treated as an interval-censored outcome).

Children who are withdrawn or lost to follow-up before the cessation of diarrhoea will be treated as censored on the day of withdrawal or loss. The primary analysis will not adjust for any covariates, but, in a second step, we will additionally explore the effect of the following covariates on the duration of diarrhoea: duration of diarrhoea prior to enrolment, prior treatment with antibiotics and prior treatment with probiotics, age and diagnosed pathogen by culture or PCR. Likewise, homogeneity of the treatment effect will be assessed according to the subgroups defined by these. The secondary end point of the time from the first dose of study medication to the start of the first 24-hour diarrhoea-free period as assessed by the parents or guardians will be analysed in the same way as the primary end point. Other secondary end points will be compared between the treatment arms on the basis of logistic regression for binary data, Poisson regression for count data, linear regression for continuous data and the lognormal accelerated failure time model for time-to-event data. The comparison of viral load outcomes for norovirus and rotavirus detected by RT-PCR will be adjusted for the participant’s baseline viral load to increase power.

The results of this trial will be reported in one or several peer-reviewed publications.

### Ethical approval

The protocol has been reviewed and approved by the Oxford Tropical Research Ethical Committee in the United Kingdom and will be submitted for review at the ethical committees of the participating hospitals in Vietnam.

## Discussion

Diarrhoeal disease in young children remains common in low- and middle-income countries such as Vietnam. The main therapy for diarrhoea consists of ORS and zinc, which are frequently supplemented with antimicrobials in severe cases with a suspected bacterial aetiology. Our recent work corroborated findings pertaining to the aetiology of diarrhoea and the accepted clinical practice of treating children hospitalised with acute watery diarrhoea in Ho Chi Minh City. We found that more than 60% of paediatric diarrhoeal cases were due to rotavirus, norovirus or a mixed infection with both (Phan Vu Tra My *et al.*, unpublished manuscript).

Probiotic products are frequently used for purposes other than those for which they were originally commercialised, namely, as medication instead of as food. The use of probiotics outside medical settings is also common in Vietnam. Probiotic brands are available inexpensively without prescriptions, and food products and formula milk are frequently supplemented with probiotics. As shown by several studies demonstrating that misidentification is the predominant cause of microbial mislabelling, there is a clear need for better quality assessment of probiotic products, specifically in regard to discrepancies between labels and the actual microbial content [28,29]. Therefore, we considered the use of a probiotic brand that is registered as a drug by the Vietnamese Ministry of Health and is produced according to international standard practices. Furthermore, we intend to ensure the consistency of the study medication by performing quality assessments at multiple time points throughout the trial.

Published studies on the effect of probiotics in treating acute watery diarrhoea in children have compared one or more of the following end points: reduction of diarrhoea duration, stool frequency, hospitalisation duration, virus in faeces and evidence of colonisation of probiotic bacteria. We will record all of these variables in an attempt to perform the most comprehensive study possible. Furthermore, we have taken into account recently published recommendations for future clinical trials using probiotics by the International Scientific Association for Probiotics and Prebiotics [30].

The nurse on duty will measure the reduction of diarrhoea duration after the start of treatment and the effect of probiotics on the total duration of diarrhoea in 12-hour intervals. As nursing staff assess patients at least every 12 hours, it is efficient to include the study assessments and data collection at those points in time. Children who participated in our previous cross-sectional study in three hospitals in Ho Chi Minh City were discharged after a median of 5 days (range, 1 to 22 days). It is difficult to collect reliable data on daily stool frequency after discharge; therefore, we have chosen to analyse stool frequency only during the first 3 days of hospitalisation. It is imperative to have a laboratory measurement for the effect of probiotics on diarrhoea; therefore, we have additionally selected the viral loads of norovirus and rotavirus in faeces as a secondary end point, as these organisms are the most common cause of acute watery diarrhoea. Norovirus and rotavirus viraemia have been described previously, and rotavirus antigenaemia has been associated with rotavirus viraemia [31,32]. We have also included an exploratory analysis on the load of norovirus and rotavirus in the blood of participants with the aim of investigating viral presence and viral load in those who test positive for norovirus or rotavirus in the stool. The ability of probiotics to colonise the intestinal tract is one of the definitions of a probiotic [33]. Colonisation by probiotics is reported to occur after several days of treatment and is reported to be transient and last for an ill-defined time period [34-37]. Hence, we have included a further secondary end point investigating the colonisation of *L. acidophilus* in faecal samples by culture and molecular techniques before treatment, at discharge and at follow-up.

Positive effects of probiotics have been reported for decades for a number of conditions. For acute diarrhoea, studies have investigated a variety of organisms, of which *Bacillus* spp., *Lactobacillus* spp., *Bifidobacterium* spp., *Streptococcus thermophilus* and *Saccharomyces cerevisiae* subtype *boulardii*, used alone or in combination, have been studied most often. These supplements have been used in a range of dosages and for differing treatment durations. Most seem to have a positive effect on the reduction of duration of diarrhoeal infections. Recently, several guidelines for probiotic and prebiotic use have been published, covering a variety of diseases, probiotic microorganisms and recommended doses based on the available evidence [38-40]. Yet, it remains unclear which probiotic should be used for the treatment of acute watery diarrhoea in our setting. We have chosen a readily available and commonly used brand in Vietnam, but other preparations may show a different effect. Furthermore, we are undecided as to what the appropriate dose should be for this indication in children. Only a limited number of trials have been published investigating dose-dependent effects of probiotics, with the suggestion that higher doses shorten diarrhoeal duration [17,41,42]. A meta-analysis by Van Niel *et al.* suggested a positive linear association between the dosage of *Lactobacillus* and the reduction in diarrhoea duration, when the results from eight studies reporting reduction in diarrhoea as the outcome, comparing *Lactobacillus* treatment to placebo, were analysed [43]. In this trial, we will use just a single dose of probiotic to compare to placebo; therefore, we will be unable to show a dose–response effect.

If results from the proposed trial demonstrate that probiotics have an effect on the primary end point of length of diarrhoeal illness, these will be used to design a larger trial to investigate the effects of different probiotics, as well as potential dose-dependent effects in more detail. In addition, we will investigate potential immunomodulating effects of a probiotic on acute watery infectious diarrhoea by measuring immune markers such as cytokines, antibodies (e.g., immunoglobulin A) and the activity of natural killer cells [44,45]. Other treatments may also influence the duration of diarrhoea, such as ORS, zinc or antimicrobials, but these have been only minimally investigated in our setting. By recording medication of the participants during hospitalisation, we will be able to investigate potential associations. Finally, in limiting our diagnostics to the most common bacterial and viral causes of diarrhoea, we will not be able to exhaustively diagnose the aetiological agents of each diarrhoeal episode. Stool samples will be stored and will be assayed retrospectively where required to detect additional pathogens.

We aim to clarify some of the reservations surrounding probiotic therapy for treating acute watery diarrhoea in hospitalised children in Vietnam. A reduction in disease duration or severity in this common disease will have a dramatic effect on both the length of stay in the hospital and the overall hospitalisation cost. This cost reduction would be beneficial for both the Vietnamese health service (which subsidises a large portion of treatment for children younger than 6 years of age) and for the families of hospitalised patients, who sacrifice a considerable amount of time and money to remain with their children in the hospital. By providing evidence of a positive, negative or absent effect of an oral preparation of *L. acidophilus* on acute watery diarrhoea, clinical practice in Vietnam might be changed to the routine prescription of probiotics to children. The ultimate aim of this change in clinical practice would be to reduce the disease burden in Vietnamese children.

## Trial status

This study protocol for a proposed randomised controlled trial has not started patient recruitment, pending the decision of local ethical committees.

## Abbreviations

CFU: colony-forming unit; CLSI: Clinical Laboratory Standards Institute; CRF: case report form; DSMB: Data Safety Monitoring Board; HCMC: Ho Chi Minh City; IL: interleukin; MOH: Ministry of Health; ORS: oral rehydration solution; RCT: randomised controlled trial; RT-PCR: reverse transcriptase polymerase chain reaction.

## Competing interests

The authors declare that they have no competing interests.

## Authors’ contributions

MK, SB and RvD conceived the study and participated in its design and coordination. MK and SB drafted the manuscript. MK, PTNT, HV, JIC, CT, MW, JF, HMT, NVVC, RvD and SB participated in the study design and submission to the Oxford Tropical Research Ethical Committee and local ethical committees. MW and CT were responsible for the sample size calculation and analysis section. PTNC, HMT, NVVC and HV participated in initiating the project. MK, SB, JIC and TTND participated in the design of the microbiological analysis plan. All authors read and approved the final manuscript.
